# Tissue and stool microbiome in pediatric inflammatory bowel disease patients: diversity differs in patients with relapsing and non-relapsing Crohn’s disease

**DOI:** 10.1186/s13099-025-00766-5

**Published:** 2025-11-15

**Authors:** Matěj Hrala, Tereza Deissová, Petr Andrla, Lenka Radová, Saša Zahornacká, Júlia Bohošová, Táňa Macháčková, Leoš Křen, Matěj Hrunka, Tereza Pinkasová, Martina Ambrozová, Juraj Bosák, Ondřej Slabý, David Šmajs, Petr Jabandžiev

**Affiliations:** 1 Department of Biology, Faculty of Medicine, Masaryk University, Brno, Czech Republic; 2 Central European Institute of Technology, Masaryk University, Brno, Czech Republic; 3 Department of Pathology, Faculty of Medicine, University Hospital Brno, Masaryk University, Brno, Czech Republic; 4 Department of Pediatrics, University Hospital Brno, Faculty of Medicine, Masaryk University, Brno, Czech Republic; 5 Center for Precision Medicine, Faculty of Medicine, University Hospital Brno, Masaryk University, Brno, Czech Republic

**Keywords:** Pediatric inflammatory bowel disease, Microbiome, Prognostic marker, Barnesiella, Crohn's disease

## Abstract

**Background:**

Inflammatory bowel diseases (IBD), including Crohn’s disease (CD) and ulcerative colitis (UC), are chronic conditions characterized by periods of clinical remission and relapse. Pediatric cases (pIBD) often have a more complicated disease course, where approximately 30% will develop a relapse within a year of diagnosis. Identifying prognostic markers for pIBD is important to optimize treatment and improve long-term outcomes. Our aim was to analyze the tissue microbiome, identify microbial prognostic markers, and validate their predictive power in non-invasive fecal samples.

**Results:**

Tissue and fecal microbiome were characterized from a prospective cohort comprising 33 therapeutically naïve pCD and 23 pUC patients, and 26 non-IBD pediatric controls, using amplicon 16S rRNA gene sequencing. Disease relapse was monitored for one year. At diagnosis, relapsing pCD patients exhibited a significantly decreased alpha diversity and altered beta diversity in tissue compared to non-relapsing pCD patients. Specific taxa were differentially abundant in relapsing pCD, with *Barnesiella* being the most depleted genus in tissue samples. Receiver Operating Characteristic (ROC) analysis identified *Barnesiella* (AUC = 0.818), *Butyricimonas*, and *Collinsella* as individual microbial tissue markers discriminating pCD relapse. Combining *Barnesiella* with the weighted Pediatric Crohn’s Disease Activity Index (wPCDAI) further enhanced the specificity and sensitivity of the ROC analysis (AUC = 0.872 in tissue, 0.852 in feces), suggesting potential for non-invasive prognostic markers from stool.

**Conclusions:**

Tissue and fecal microbial markers can predict relapse in pCD patients with high prognostic power, providing a basis for precision medicine and personalized treatment strategies in pIBD.

**Supplementary Information:**

The online version contains supplementary material available at 10.1186/s13099-025-00766-5.

## Background

Inflammatory bowel diseases (IBD) are a group of chronic, relapsing inflammatory conditions of the gastrointestinal tract with increasing prevalence in the population [1]. Two major subtypes, Crohn’s disease (CD) and ulcerative colitis (UC), exhibit distinct clinical and pathological features. The pediatric cases (pIBD) are frequently associated with a more complicated disease course compared to adult-onset IBD [2]. The prevalence of pediatric IBD varies across studies but is generally increasing, with the greatest numbers in Western Asia and Western Europe (up to 21.6 and 17.4 cases per 100,000 person-years, respectively) [3].

The etiology of pIBD remains elusive, but it involves a complex interplay of genetic, environmental, and microbial factors. The disruptions in the gut microbiota composition and function are implicated in the pathogenesis of IBD in both pediatric and adult patients and may involve the loss of certain taxa of microorganisms and a reduction in the overall alpha diversity of the microbial community. However, whether dysbiosis is a cause or a consequence of the inflammatory process remains unclear. Prior research on tissue samples has revealed that pIBD patients consistently exhibit lower gut microbiome diversity compared to healthy individuals. Furthermore, these studies report a characteristic shift in microbial composition, marked by decreased levels of Firmicutes, and the genera *Faecalibacterium*, and *Bifidobacterium*, and increased levels of *Escherichia* or *Veillonella* in the gut microbiota of pIBD patients [4–6].

Current diagnostic approaches rely on a combination of clinical, endoscopic, and histological assessments. Stool analysis provides a non-invasive assessment of gut microbiota composition and presence of inflammatory markers (i.e., fecal calprotectin), while tissue biopsies obtained through endoscopy offer valuable information about the extent and severity of intestinal inflammation and histological features characteristic of each IBD subtype. Despite significant progress in the management of pediatric IBD, about one-third of patients develop a relapse within a year of diagnosis [7]. A reliable prognostic marker that would predict the course of the disease at the time of diagnosis would help to set a treatment strategy and reduce the incidence of relapse.

In this study, we characterized the tissue and fecal microbiome of a therapeutically naïve cohort of 33 pCD patients, 23 pUC patients, and 26 non-IBD pediatric controls. The variable outcomes observed within these pIBD groups allowed for the identification of potential prognostic markers associated with pCD relapse.

## Materials and methods

### Study participants and sample collection, and ethical statement

This study was conducted as a mono-centric, prospective case-control study, approved by the Ethics Committee of the University Hospital Brno (Approval Code: 27–100620/EK). The sample collection took place at the Department of Pediatrics, University Hospital Brno, Czech Republic. In adherence to the Helsinki Declaration, written informed consent was obtained from the parents of all participating children before any study procedures.

The case group consisted of children diagnosed with pediatric inflammatory bowel disease (pIBD, *N* = 56) by experienced pediatric gastroenterologists, based on the ESPGHAN revised Porto criteria. Within the pIBD cohort, children were identified as having either pediatric Crohn’s disease (pCD, *N* = 33) or pediatric ulcerative colitis (pUC, *N* = 23). IBD unclassified or other nonspecific cases were excluded. The control group (non-IBD, *N* = 26) comprised children undergoing endoscopy because of abdominal pain, and pIBD was excluded using laboratory, radiological, endoscopic, and histological evaluations. Both pIBD and non-IBD participants taking antibiotics during the examination period were excluded from the study.

Samples were collected from all participants as follows. Two fresh-frozen tissue biopsies were obtained from three gut regions: the terminal ileum (TEI), caecum-ascendens (right hemicolon, RHC), and rectosigmoideum (left hemicolon, LHC). One biopsy from each region was evaluated by an experienced pathologist at the Department of Pathology, University Hospital Brno, Masaryk University, to confirm or exclude microscopic inflammation. The second biopsy was preserved in RNAlater™ Stabilization Solution (Invitrogen™, Thermo Fisher Scientific, Waltham, MA, USA) and stored at − 80 °C for further research. Additionally, stool samples were collected in ESwab tubes with Amies medium (Copan, Ave, Murrieta) and stored at − 80 °C until subsequent analyses. Moreover, these clinical data were collected: disease activity indices (weighted Pediatric Crohn’s Disease Activity Index [wPCDAI] and Pediatric Ulcerative Colitis Activity Index [PUCAI]), hemoglobin levels, C-reactive protein levels, and fecal calprotectin levels. To assess clinical disease activity over time, disease status was evaluated at three, six, and twelve months after the initial diagnosis using wPCDAI or PUCAI scores and fecal calprotectin (FCP) level. Additionally, disease relapse within 12 months post-diagnosis was monitored as a prognostic indicator of disease severity and/or treatment response [8]. Relapse in pIBD patients was defined as a recurrence of symptoms with a PUCAI score >10, a wPCDAI score >12.5, or an FCP level >300 µg/g [9–11]. During relapse, patients were thoroughly investigated to rule out infectious causes. Moreover, the dietary characteristics for each patient were recorded (Supplementary Table S1).

## Sample DNA extraction

A biopsy sample stored in RNAlater™ Stabilization Solution (Invitrogen™, Thermo Fisher Scientific, Waltham, MA, USA) was thawed on ice, and 1 × 1 mm section was aseptically separated. The resulting tissue fragment was placed into ZR BashingBead™ Lysis tubes (0.1 & 0.5 mm) containing 750 µL of ZymoBIOMICS™ Lysis Solution, which is part of the ZymoBIOMICS™ DNA Miniprep Kit (Zymo Research, Irvine, USA). The stool sample stored in ESwab tubes with Amies medium (Copan, Ave, Murrieta, USA) was thawed on ice, homogenized by vortexing, and 200 µL was added to the ZR BashingBead™ Lysis tubes (0.1 & 0.5 mm), along with 550 µL of ZymoBIOMICS™ Lysis Solution. In addition, ZymoBIOMICS Fecal Reference with TruMatrix™ Technology and ZymoBIOMICS Gut Microbiome Standard (both Zymo Research, Irvine, USA) were used as positive controls for both DNA isolation and sequencing. The taxonomic composition from the sequencing controls correlated with the expected composition (Spearman’s rank correlation: r_s_ = 0.799 and r_s_ = 0.939, respectively; both *p* < 0.001). Microbial DNA-free water (Sigma-Aldrich, Burlington, Massachusetts, USA) was used as a negative control. To each sample, 20 µL of Proteinase K (20 mg/mL stock concentration; GEXPRK01-I5, Elisabeth Pharmacon, Brno, Czech Republic) was added, and the sample was incubated for 1 h at 55 °C and 600 RPM in the Thermomixer comfort. Homogenization was performed 4 times for 60 s at 9000 RPM, with a 2-minute pause between cycles, using the Precellys Evolution homogenizer (Bertin Technologies SAS, France). The subsequent steps of the extraction followed the manufacturer’s recommendations for the ZymoBIOMICS™ DNA Miniprep Kit (Zymo Research, Irvine, USA). The quality of the isolated DNA was checked using a NanoDrop™ 2000/2000c spectrophotometer. The quantity of extracted DNA was measured using the QuantiFluor^®^ dsDNA System, and the isolated DNA was stored at − 20 °C until further analysis.

### 16S rRNA sequencing library preparation

Isolated DNA was thawed at room temperature and amplified using a double-round PCR reaction. For the 16S rRNA library preparation, the Quick-16S™ Primer Set V1-V2 (Zymo Research, Irvine, USA) was employed to target the variable V1-V2 regions of the 16S rRNA gene. The first PCR amplification was conducted in a 10 µL reaction volume containing 5 µL of KAPA2G Robust HotStart ReadyMix PCR Kit 2x (Sigma-Aldrich, Burlington, Massachusetts, USA), 2 µL of Quick-16S™ Primer Set V1-V2 (Zymo Research, Irvine, USA) for 16S rRNA, and 2 µL of template DNA. For 16S rRNA library preparation, genomic DNA isolated from stool samples was diluted to a 5 ng/µL concentration, while undiluted DNA samples were utilized in other cases. The thermal cycling profile for the initial round of PCR consisted of an initial denaturation at 95 °C for 3 min, followed by 25 cycles of denaturation at 95 °C for 15 s, annealing at 55 °C for 30 s, and extension at 72 °C for 40 s, with a final extension step at 72 °C for 1 min. The amplicon product from this reaction (10 µL) was then purified using 4 µL of ExoSAP-IT™ PCR Product Cleanup Reagent (Applied Biosystems™, Waltham, Massachusetts, USA), with the purification process involving incubation at 37 °C for 15 min followed by enzyme deactivation at 80 °C for 15 min. In the second round of PCR, dual i7 and i5 Nextera DNA indexes (Illumina, San Diego, CA, USA) were added for indexing amplicons. The reaction volume was 20 µL and included 10 µL of KAPA2G Robust HotStart ReadyMix PCR Kit 2x (Sigma-Aldrich, Burlington, Massachusetts, USA), 4 µL of dual i7 and i5 index primers, 4 µL of microbial DNA-free water (Sigma-Aldrich, Burlington, Massachusetts, USA), and 2 µL of the purified DNA amplicon product from the first PCR. The thermal cycling profile started with an initial denaturation at 95 °C for 3 min, followed by 7 cycles of denaturation at 95 °C for 15 s, annealing at 55 °C for 30 s, and extension at 72 °C for 40 s, with a final extension at 72 °C for 1 min. Indexed DNA product lengths were checked on a 1% agarose gel using a Uvitec instrument. Indexed PCR products were then pooled and purified using SPRIselect beads (Beckman Coulter, California, USA). The concentration of the sequence library pool was assessed with the QuantiFluor^®^ dsDNA System (Promega, Madison, Wisconsin, USA), while its length was determined using the Agilent 2200 TapeStation system in combination with the Agilent High Sensitivity D1000 ScreenTape System (Agilent Technologies). Preparation of the library for sequencing followed the protocol for Denature and Dilute Libraries for the MiSeq system. The finalized library was spiked with 30% PhiX Control v3. Paired-end sequencing was then conducted using the MiSeq Sequencing System with MiSeq Reagent Kit v3 (600 cycles, Illumina, San Diego, CA, USA), generating reads with a length of 2 × 300 bp.

## Bioinformatic analysis

Raw paired-end 16S rRNA gene sequences underwent quality control using FastQC (v0.11.5) [12] and FastQ Screen (v0.15.3) [13]. Adapter trimming and quality filtering (Q ≥ 25, minimum length 40 bp) were performed with Fastp (v0.20.1) [14]. Host reads were subsequently removed by alignment against the GRCh38 reference genome using Bowtie2 (v2.4.2) [15]. Denoising, chimera removal, and amplicon sequence variant (ASV) inference were performed using DADA2 implemented via QIIME 2 (v2024.5). Each sequencing run was processed separately for error correction, read merging, and chimera detection. Taxonomic assignment of representative ASVs obtained by DADA2 was performed using the Naïve Bayes classifier [16] and the SILVA database v138 (99% identity) [17]. Contaminant ASVs were identified and removed using Decontam (v1.12.0), applying the prevalence method with negative controls indicated in the metadata. Taxa with contamination scores exceeding 0.1 were discarded. The ASV table was further filtered by minimum abundance (≥ 10 reads across all samples) and minimum prevalence (≥ 2 samples).

Alpha diversity metrics (Shannon, Simpson, observed ASVs) and beta diversity metrics (Bray–Curtis, Jaccard, unweighted UniFrac, and weighted UniFrac) were calculated on rarefied feature Tables (10,000 reads/sample). Group differences in alpha diversity were assessed using the Kruskal–Wallis test, while beta diversity differences were tested using PERMANOVA. Principal coordinates analysis (PCoA) plots were constructed using beta diversity metrics to visualize sample clustering patterns. Differential abundance analysis was performed using ANCOM-BC, a QIIME 2 plugin (v2024.5), with a significance threshold of 0.05.

## Logistic regression models, receiver operating characteristic (ROC) curve generation and analysis

The multivariate logistic regression models were applied in order to identify a combination of predictors that hold prognostic value for disease outcome. The best model for each outcome was determined through bidirectional stepwise selection, which iteratively added or removed microbial taxa to minimize the Akaike information criterion (AIC). Predictive performance was evaluated through the sensitivity and specificity of the models, summarized in ROC curves (https://cran.r-project.org/web/packages/boot/index.html). A risk score formula for predicting individual outcomes was developed as a linear combination of microbial abundance levels, weighted by regression coefficients derived from the logistic regression model. Patients were stratified into high-risk and low-risk groups based on a threshold designed as the value maximizing the sum of sensitivity and specificity. Confidence intervals (CIs) for sensitivity, specificity, accuracy, positive predictive value (PPV), and negative predictive value (NPV) for the threshold were calculated using Wilson’s formula with 2,000 bootstrap replicates of the ROC curve [18, 19]. An AUC greater than 0.7 was considered the threshold for acceptable markers [20].

### Statistical analysis

Data analyses were performed in R [21]. Statistical comparisons were performed using the Kruskal-Wallis test for alpha diversity, PERMANOVA for beta diversity, and ANCOM-BC for taxon-level comparisons. P-values lower than 0.05 were considered statistically significant. The two-tailed Fisher’s exact test was used for comparison of diet preferences of patients/controls. An unpaired two-tailed Student’s t test was used for statistical comparisons of clinical parameters. To evaluate the accuracy of taxonomic profiling from sequencing and isolation controls, Spearman’s rank correlation coefficient (rₛ) was calculated between the observed and expected relative abundances of taxa. Correlation analyses were performed using GraphPad Prism.

## Results

### Cohort characterization

Microbiome analyses were performed on a clinically characterized prospective children’s cohorts comprising newly diagnosed, treatment-naïve pIBD patients with active pCD or pUC and non-IBD controls (see Table 1). No significant differences in dietary restrictions or comorbidities were found between the matched groups, with the exception of a higher incidence of primary sclerosing cholangitis in the pUC group compared to the non-IBD controls (Table 1; Supplementary Table S1). Intestinal tissues with confirmed microscopic inflammation from pIBD patients and non-inflammatory tissues from non-IBD children were analyzed. To account for the potential impact of microbial composition across different intestinal sites, tissue origins were largely matched between pIBD and non-IBD groups (see Table 1). Specifically, non-IBD samples were obtained from the terminal ileum (TEI) to broadly match pCD patients and the left hemicolon (LHC) to broadly match pUC patients. pIBD patient samples were always collected from inflamed sites: TEI or right hemicolon (RHC) for pCD, and LHC or RHC for pUC. In addition, we performed 16S rDNA amplicon sequencing analysis with stool samples of pIBD and non-IBD children.

**Table 1:** Characteristics of the study cohort

Group	non-IBD	pCD		pUC	
**Participants**, *N*	26	33		23	
**Age**, median (min, max)	17 (6, 18)	14 (6, 18)		14 (6, 17)	
**Sex**, boys: girls	8:18	18:15		16:7	
**Diet**, %			p-value*		p-value*
without restriction	61.5	66.7	0.7864	43.5	0.2579
lactose-free	34.6	27.3	0.5801	52.2	0.2570
low-fiber	0.0	6.1	0.4985	4.3	0.4694
gluten-free	3.8	0.0	0.4407	0.0	1.000
**Comorbidities**, %			p-value*		p-value*
Atopic dermatitis	7.7	9.1	1.000	4.3	1.000
Bronchial asthma	7.7	12.1	0.6852	0.0	0.4915
Primary sclerosing cholangitis	0.0	0.0	1.000	17.4	0.0418
Allergies	30.8	30.3	1.000	13.0	0.1805
Others^#^	30.8	12.1	0.1068	13.0	0.1805
**Sampled area**, *N*	26 (TEI to pCD**) and 26 (LHC to pUC**)	25 (TEI) and 8 (RHC)		19 (LHC) and 4 (RHC)	
**Disease location** (Paris classification), %					
L1/E1	-	12.1	-	8.7	-
L2/E2	-	18.2	-	8.7	-
L3/E3	-	45.5	-	4.3	-
L3 + L4/E4	-	24.2	-	78.3	-
**wPCDAI/PUCAI**, mean					
initial	-	23.0	-	27.2	-
3 months	-	10.5	-	4.8	-
12 months	-	5.3	-	4.2	-
**FCP** (µg/g), mean					
initial	-	729.5	-	652.0	-
3 months	-	597.8	-	371.4	-
12 months	-	352.2	-	314.9	-

*P-value denotes differences between the non-IBD control group and either the pCD or pUC group; the two-tailed Fisher’s exact test was used to calculate statistical significance. **Two tissue samples were taken from different areas in non-IBD controls; LHC = left hemicolon, RHC = right hemicolon, TEI = terminal ileum. ^#^See Supplementary Table S1 for more details

**Fig. 1:**
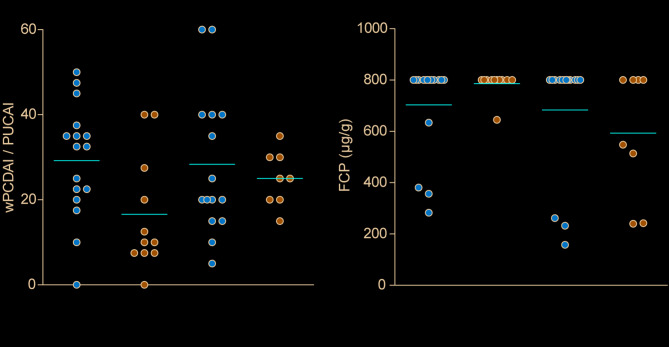
wPCDAI/PUCAI and FCP characteristics of patients with and without relapse at the time of diagnosis. Each dot represents an individual patient. Panel (**A**) shows wPCDAI for pCD patients and PUCAI for pUC patients, while panel (**B**) shows FCP values. Both panels are categorized by relapse status. wPCDAI/PUCAI and FCP were determined at initial diagnosis, followed by wPCDAI/PUCAI at 3, 6, and 12 months, and FCP at 3 and 12 months. All data from every time point are available in Supplementary Table S1. Patient numbers vary depending on the participation in follow-up testing (numbers shown). Moreover, six pCD patients (out of 33) were excluded from these categories because they did not achieve remission within 12 months. Red bar; mean

pCD patients with relapse (return of symptoms after remission) within 12-month period, requiring treatment escalation, exhibited significantly higher wPCDAI values at the time of diagnosis (Fig. 1A). In pUC patients with relapse, no statistically significant differences were observed for PUCAI and FCP at the time of diagnosis (Fig. 1A, B). Complete data for wPCDAI/PUCAI, FCP, CRP, hemoglobin, and initial therapy are presented in Supplementary Table S1.

## Tissue microbiome differs between pIBD patients and non-IBD controls

**Fig. 2:**
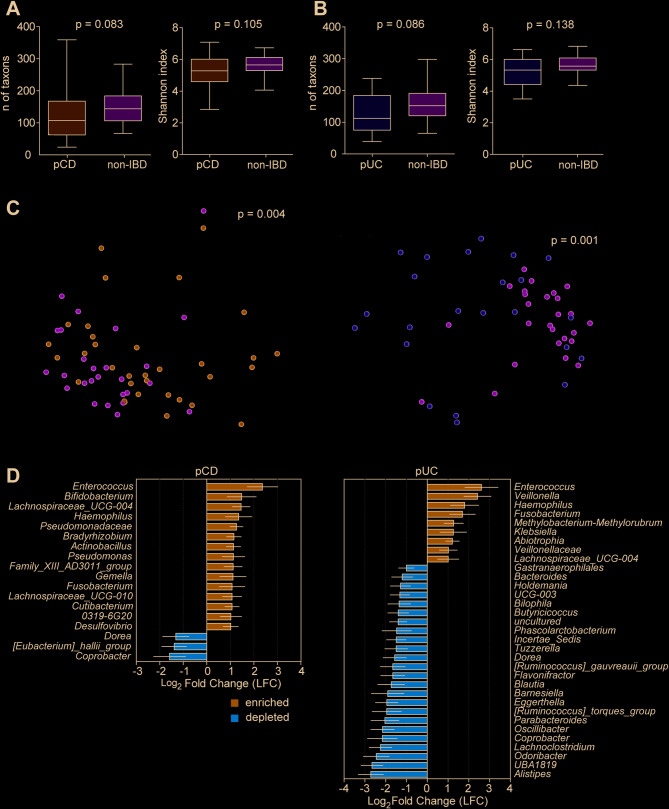
Microbial diversity and composition in tissue samples from patients with pCD (*N* = 33), pUC (*N* = 23), and non-IBD controls (*N* = 26). Taxon richness and the Shannon index (alpha diversity) tended to be lower in pCD (**A**) and pUC (**B**) compared to non-IBD controls. **(C)** Beta diversity, assessed by unweighted UniFrac, showed significant separation between pIBD patients and non-IBD controls (pCD, blue; pUC, yellow; non-IBD, green). (**D**) Genera significantly enriched (Log_2_ Fold Change (LFC) > 1) and depleted (LFC < −1; *p* < 0.05) in pCD (left) and pUC (right) groups when compared to controls. Box plots: median, interquartile range (box), min-max range (whiskers). Bars represent the mean of taxa, with error bars showing 95% confidence intervals. Statistical comparisons were performed using the Kruskal-Wallis test for alpha diversity, PERMANOVA for beta diversity, and ANCOM-BC for taxon-level comparisons. A complete list of significantly different taxa is shown in Supplementary Table S2

The diversity of microbial community composition was evaluated in tissue samples from pIBD patients and a control group. Alpha diversity analysis revealed no statistically significant differences in taxon richness between patient and control groups. However, both the pCD and pUC groups showed a trend towards decreased richness (*p* = 0.083 and 0.086, respectively; Fig. 2A, B) and towards lower Shannon index values compared to controls (*p* = 0.105 and 0.138, respectively; Fig. 2A, B). Beta diversity analysis showed a clear difference between the microbial communities of controls and patients with pCD (*p* = 0.004) and pUC (*p* = 0.001; Fig. 2C). Based on differential abundance analysis, in pCD tissue samples, 15 taxa were enriched and three were depleted. In pUC samples, nine taxa were enriched and 24 were depleted (Fig. 2D). At the genus level, *Enterococcus* was the most significantly enriched taxon in both pCD and pUC patients. The most depleted taxa were *Coprobacter* in pCD and *Alistipes* in pUC (Fig. 2D). A complete list of abundances of significantly different taxa is shown in Supplementary Table S2.

### Microbial tissue diversity and taxonomic profiles differentiate relapsing and non-relapsing pCD

**Fig. 3:**
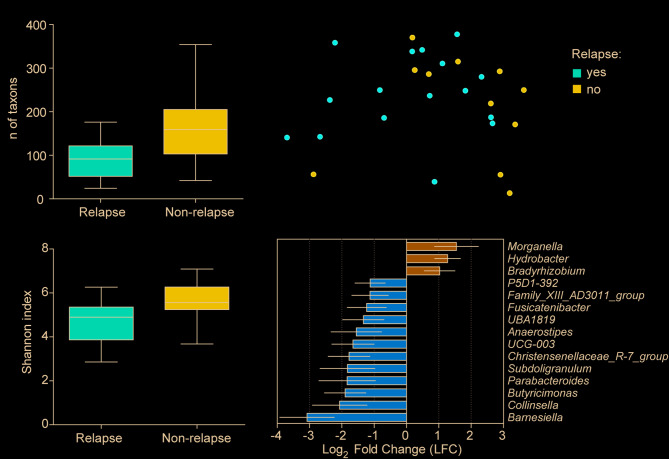
Microbial diversity and composition in tissue samples of pCD patients with relapse (*N* = 16) when compared to non-relapsing patients (*N* = 11). (**A**) Taxon richness and (**B**) Shannon index were significantly lower in pCD patients with relapse. (**C**) Beta diversity, assessed by unweighted UniFrac, showed significant separation between pCD patients with and without relapse. (**D**) Genera abundance significantly enriched (LFC > 1; *p* < 0.05) and depleted (LFC < −1; *p* < 0.05) in pCD patients with relapse. Six pCD patients (out of 33) were excluded from these categories because they did not achieve remission within 12 months. Box plots: median, interquartile range (box), min-max range (whiskers). Bars represent the mean of taxa, with error bars showing 95% confidence intervals. Statistical comparisons were performed using the Kruskal-Wallis test for alpha diversity, PERMANOVA for beta diversity, and ANCOM-BC for taxon-level comparisons

Patients experiencing disease improvement followed by relapse requiring treatment escalation within one year of treatment initiation were classified as having disease relapse (*N* = 16 for pCD). Relapsing pCD patients exhibited decreased taxon richness (*p* = 0.023) and diversity (Shannon index; *p* = 0.020; Fig. 3A, B). Beta diversity analysis also revealed significant differences between these two groups (*p* = 0.034; Fig. 3C). At the genus level, three taxa were significantly enriched (the most enriched being *Morganella*), while 12 taxa were significantly depleted (the most depleted being *Barnesiella*; Fig. 3D).

In contrast, no significant differences in alpha or beta diversity were found between relapsing and non-relapsing pUC patients, the significantly different taxa are shown in Supplementary Figure S1.

### Identification of relapse indicators in Crohn’s disease patients

**Fig. 4:**
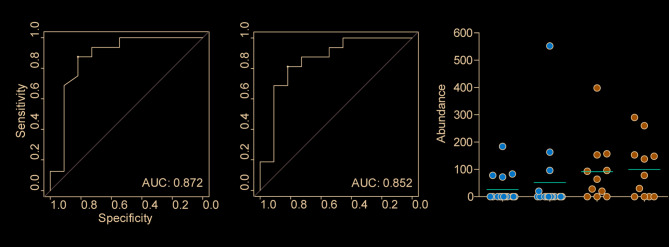
**(A)** Combining two parameters in stool samples yielded comparable AUC values in ROC analysis to those obtained with tissue, suggesting their potential as non-invasive markers for pCD relapse prognosis. **(B)** Paired analysis of *Barnesiella* abundance in tissue and stool samples from individual pCD patients with and without relapse. This taxon shows potential as prognostic marker for relapse in pCD, as it differs between relapse groups but remain comparable between sample types (tissue, stool). Red bar; mean

Clinical disease activity, as measured by the weighted Pediatric Crohn’s Disease Activity Index (wPCDAI), or alpha diversity (richness and Shannon index), discriminated between pCD patients with and without relapse in tissue samples, with area under the curve (AUC) values ranging from 0.744 to 0.767 (Supplementary Table S3). The abundance levels of individual microbial taxa in tissue samples were also assessed for their ability to differentiate between pCD patients with and without relapse using ROC analysis. This analysis revealed that the AUC for the three taxa with the best discriminatory power (*Barnesiella*, *Butyricimonas*, and *Collinsella*) was higher than 0.773, with *Barnesiella* exhibiting the highest AUC (0.818). All of these taxa were significantly depleted in pCD patients experiencing relapses, conversely none of the significantly enriched taxa (Fig. 3D) showed discriminatory power higher than AUC = 0.7 in ROC analysis. A complete list of AUC values is shown in Supplementary Table S3. Furthermore, the combination of wPCDAI and *Barnesiella* abundance achieved an improved AUC of 0.872 (Fig. 4A).

To identify the most suitable indicators of relapse in pCD patients with the highest discrimination power, the multivariate logistic regression was performed. The predictor model can predict pCD relapse within 12 months after the diagnosis with AUC = 1 (cut-off = 0.0351, sensitivity = 1, specificity = 1, accuracy = 1, positive predictive value = 1, negative predictive value = 1) with risk formula = −527.856309 + 0.124793 * *Morganella* + 102.311292 * *Hydrobacter* + 27.816742 * *Bradyrhizobium* − 26.013712 * *Family_XIII_AD3011_group* − 13.612790 * *Fusicatenibacter* − 6.341383 * *Butyricimonas* + 12.154157 * Richness. The corresponding ROC curve for this model can be found in Supplementary Figure S2.

### Verification of tissue relapse indicators in pediatric Crohn’s disease in stool samples

Identical microbiome analyses, as employed for tissue samples, were applied to stool samples obtained from the same patients diagnosed with pCD and pUC. A panel with alpha diversity, beta diversity and taxonomical differences results can be found in Supplementary Figure S3. Considering pCD patients with and without relapse, analysis of stool samples revealed a trend towards decreased alpha diversity (Supplementary Figure S4) in relapsing patients compared to non-relapsing ones, consistent with tissue sample findings, although this trend was not statistically significant. Differences in beta diversity were statistically significant only for the Jaccard distance. At the genus level, *Barnesiella* and the temporarily assigned *UCG-003* taxon from the family Oscillospiraceae were the genera significantly depleted in stool of pCD patients experiencing relapse that also corresponded to depletion observed in tissue analysis (Supplementary Figure S4). To explore the feasibility of using these parameters as non-invasive prognostic markers derived from stool, ROC curves were generated with stool samples, similar to the analysis conducted on tissue. *Barnesiella* and the temporarily assigned *UCG-003* taxon exhibited rather modest AUC values of 0.696 and 0.693, respectively. Given that *Barnesiella* demonstrated a more significant depletion in tissue samples compared to *UCG-003*, its individual value as a marker is more robust. Despite the modest individual performance of *Barnesiella* in stool, the combination of *Barnesiella* abundance and wPCDAI showed notably improved and comparable predictive power in both stool (AUC = 0.852) and tissue (AUC = 0.872) samples (Fig. 4A). A consistent depletion trend of *Barnesiella* abundance in relapsing pCD patients across both tissue and fecal microbiomes is specifically illustrated in Fig. 4B.

The same multivariate logistic regression model as for tissue samples was also applied to stool samples, with detailed results presented in the Supplementary Figure S2, where the AUC for the model reached 1.

## Discussion

The highly variable clinical course of inflammatory bowel diseases (IBD) presents a major challenge in pediatric patient diagnosis and management [22]. Moreover, the limited correlation observed between clinical activity, biological markers, and endoscopic findings in IBD patients complicates accurate assessment of disease severity [23]. Prediction of pIBD prognosis plays an important role in the clinical decisions and disease management and wPCDAI/PUCAI and FCP are currently used by clinicians as non-invasive markers for pIBD prognosis prediction [24, 25]. In the present study, we have observed that pCD patients who relapsed within one year after diagnosis had a significantly higher wPCDAI value at diagnosis. Moreover, the clinical scoring index wPCDAI demonstrated a moderate discriminatory power (AUC = 0.744) when considering that it offers a non-invasive approach prognosis assessment of pCD patients.

Given the very limited number of available pIBD tissue microbiome studies [26, 27], this research, encompassing 33 pCD patients, 23 pUC patients, and 26 non-IBD controls, makes an important contribution. In addition, the microbiome composition of pIBD patients remains an unexplored area for the identification of prognostic markers. This study found a decreased overall alpha diversity in the tissue microbiome of pIBD patients, consistent with the previous findings in pCD [26] and pUC [28]. Similarly, the determined beta-diversity differences observed here align with other studies [29, 30]. While the specific enriched and depleted taxa differed between pCD and pUC in our cohort, certain taxa, such as *Enterococcus*, were enriched in both, which has been previously reported [31].

To identify the most effective prognostic markers for pCD in tissue microbiome, we employed the ROC curve analysis. Our results revealed that specific microbial taxa demonstrated significant discriminatory potential between pCD patients with and without relapse. Notably, *Barnesiella* (AUC = 0.818), *Butyricimonas* (AUC = 0.790), and *Collinsella* (AUC = 0.773) emerged as promising markers, with all three taxa showing depletion in pCD patients experiencing relapse. Conversely, none of the taxa significantly enriched in pCD patients with relapse exceeded AUC threshold of 0.7, suggesting that the depletion of certain microbial members might be a stronger indicator of a poorer prognosis in pCD. Previous attempts to identify microbial markers for the prediction of pIBD severity include the work by Meij et al. (2018) [32], which found that reduced *Alistipes finegoldii* and *A. putredinis* among pIBD patients could be a good indicator of the disease severity (AUC = 0.87). The study by Wang et al. (2021) [33] proposed a disease severity prediction based on eleven bacterial genera (11-OTU stool model, AUC = 0.84–0.88). In contrast, the overall stability of the microbiome in pUC patients, irrespective of their disease activity status [34], suggests why a predictive marker for relapse was not found in this study. As suggested by our study, the genus *Barnesiella* is a promising microbial marker for predicting pCD disease prognosis. The depleted abundance of *Barnesiella* in pCD patients with relapse aligns with the findings reported by Alipour et al. (2016) [28], who showed a decreased abundance of the Barnesiellaceae family in tissue samples from both pCD and pUC patients compared to non-IBD children. Chen et al. (2022) [35] identified the genus *Barnesiella* as a potential marker, among five others, for predicting positive patient responsiveness to adalimumab, a commonly used IBD treatment that targets and neutralizes TNF-alpha. Moreover, Vestergaard et al. (2024) [36] demonstrated a lower abundance of *Barnesiella* in individuals with IBD as well as decreased abundance of *UCG-003* (family Oscillospiraceae), which they noted as the first such observation in the literature [36]. Consistent with these findings, our study also found *UCG-003* to be significantly depleted in both the tissue and fecal microbiomes of pCD patients experiencing relapse. The significance of *Barnesiella* depletion extends beyond IBD, as it has been identified in a meta-analysis as a more universal microbial marker for additional intestinal diseases, including colorectal cancer [37]. A higher abundance of *Barnesiella* in healthy individuals compared to those with intestinal diseases suggests its correlation with intestinal health [37]. Moreover, the study of Mancabelli et al. (2017) [37] showed a high abundance of Christensenellaceae R-7 group in healthy individuals, and this taxon was found to be depleted in pCD patients with relapse in our study (with an AUC of 0.733 determined in this study). Although the precise role bacterial genera associated with microbiome of healthy individuals is not clear, *Barnesiella* may be involved in degradation of complex carbohydrates, facilitating cross-feeding interactions with other beneficial gut microbes often reduced in IBD [38]. 

In our study, alpha and beta diversity significantly differed between relapsing and non-relapsing pCD patients in tissue samples, however, this distinction was not evident in stool samples. A similar finding was reported by Gevers et al. (2014) [26] that suggested a higher diagnostic power in tissue samples compared to stool samples. However, other authors including Wang et al. (2016) [39] found results that are comparable between tissue and stool samples. A depletion of *Barnesiella* genus found in this work was observed in both tissue and stool samples from pCD patients experiencing a relapse. Confirming this depletion in both sample types is critical for establishing a reliable prognostic marker. Moreover, this finding is important since the identification of prognosis markers of pCD patients in stool samples could avoid repeated intestinal biopsies. The *Barnesiella* depletion showed strong standalone predictive performance for relapse within 12 months in tissue (AUC = 0.818), but poor performance in stool (AUC = 0.696). However, this prediction was considerably stronger when combined with wPCDAI (AUC = 0.872 in tissue and 0.852 in stool). This combined performance is substantially higher than the wPCDAI standalone score (AUC = 0.744). Predictors with an AUC >0.8 are generally considered excellent markers [20]. At the individual patient level, *Barnesiella* in tissue sample microbiome was completely absent in 12 out of 16 relapsing patients and only in 3 out of 11 non-relapsing patients (Fig. 4B), suggesting the presence of both qualitative and quantitative differences between pCD and controls. The abundance of *Barnesiella* in the human microbiota appears to be a promising prognostic marker, but establishing its clear clinical threshold may be challenging, particularly for tests relying on real-time PCR detection. Setting this threshold remains a goal for future research. In this context, determination of complete 16S rRNA gene microbiome analysis, including determination of microbiome diversity, composition, and taxon richness, may offer a more reliably interpretable alternative, as in this case, *Barnesiella* abundance thresholds could be defined based on its percentage within the total microbiome and could be determined together with microbiome richness.

Unlike most other studies that primarily analyze the fecal microbiome, a key strength of this study lies in the simultaneous analysis of both tissue and stool samples obtained from the same pIBD patients. This unique approach enabled us to establish microbiome characteristics directly from tissue, which better reflects the patient’s intestinal environment, and then directly correlate these findings with corresponding stool samples. However, the limitations of this study include a relatively small number of patients and non-IBD controls within the cohorts, imperfectly matched biopsy areas between patient and non-IBD control samples, and the exclusion of patients with persistent active disease after induction therapy from relapse group, which may have introduced selection bias and potentially limits the generalizability of our findings to the broader pediatric IBD population.

## Conclusion

Our findings revealed the gut microbiome as a non-invasive source of prognostic markers in pCD. Alterations in the abundance of certain bacterial taxa, particularly the depletion of *Barnesiella*, appear to predict disease relapse. While further research is needed to validate these microbial signatures in pediatric and adult populations and integrate them with clinical parameters like wPCDAI, our study suggests that assessing the fecal microbiome holds promise for improving prognostic evaluation and guidance of clinical management in pCD.

## Supplementary Information

Supplementary Material 1

Supplementary Material 2

Supplementary Material 3

Supplementary Material 4

Supplementary Material 5

Supplementary Material 6

Supplementary Material 7

## Data Availability

The authors declare that all other data supporting the findings of this study are available within the article and its Supplementary material files, or are available from authors upon request.

## References

[1] Agrawal M, Jess T. Implications of the changing epidemiology of inflammatory bowel disease in a changing world. United Eur Gastroenterol J [Internet]. 2022 [cited 2025 Jun 13];10:1113–20. Available from: https://pubmed.ncbi.nlm.nih.gov/36251359/10.1002/ueg2.12317 PMC9752308 36251359

[2] Pigneur B, Seksik P, Viola S, Viala J, Beaugerie L, Girardet JP et al. Natural history of Crohn’s disease: Comparison between childhood- and adult-onset disease. Inflamm Bowel Dis [Internet]. 2010 [cited 2025 Jun 13];16:953–61. Available from: https://pubmed.ncbi.nlm.nih.gov/19834970/10.1002/ibd.21152 19834970

[3] Kuenzig ME, Fung SG, Marderfeld L, Mak JWY, Kaplan GG, Ng SC et al. Twenty-first Century Trends in the Global Epidemiology of Pediatric-Onset Inflammatory Bowel Disease: Systematic Review. Gastroenterology [Internet]. 2022 [cited 2025 Jun 13];162:1147–1159.e4. Available from: https://pubmed.ncbi.nlm.nih.gov/34995526/10.1053/j.gastro.2021.12.282 34995526

[4] Haberman Y, Tickle TL, Dexheimer PJ, Kim MO, Tang D, Karns R et al. Pediatric Crohn disease patients exhibit specific ileal transcriptome and microbiome signature. J Clin Invest [Internet]. 2014 [cited 2025 Jun 13];124:3617–33. Available from: https://pubmed.ncbi.nlm.nih.gov/25003194/10.1172/JCI75436 PMC4109533 25003194

[5] Wang F, Kaplan JL, Gold BD, Bhasin MK, Ward NL, Kellermayer R et al. Detecting Microbial Dysbiosis Associated with Pediatric Crohn Disease Despite the High Variability of the Gut Microbiota. Cell Rep [Internet]. 2016 [cited 2025 Jun 13];14:945–55. Available from: https://pubmed.ncbi.nlm.nih.gov/26804920/10.1016/j.celrep.2015.12.088 PMC4740235 26804920

[6] Wu P, Wu B, Zhuang Z, Liu J, Hong L, Ma B et al. Intestinal mucosal and fecal microbiota profiles in Crohn’s disease in Chinese children. Med Microecol [Internet]. 2023 [cited 2025 Jun 13];15:100071. Available from: https://www.sciencedirect.com/science/article/pii/S2590097822000210

[7] Chauhan N, Khan HH, Kumar S, Lyons H. Clinical Variables as Predictors of First Relapse in Pediatric Crohn’s Disease. Cureus [Internet]. 2019 [cited 2025 Jun 13];11. Available from: https://pubmed.ncbi.nlm.nih.gov/31467814/10.7759/cureus.4980 PMC6706256 31467814

[8] Kato J, Yoshida T, Hiraoka S. Prediction of treatment outcome and relapse in inflammatory bowel disease. Expert Rev Clin Immunol [Internet]. 2019 [cited 2025 Jun 13];15:667–77. Available from: https://pubmed.ncbi.nlm.nih.gov/30873890/10.1080/1744666X.2019.1593140 30873890

[9] Turner D, Otley AR, Mack D, Hyams J, de Bruijne J, Uusoue K et al. Development, Validation, and Evaluation of a Pediatric Ulcerative Colitis Activity Index: A Prospective Multicenter Study. Gastroenterology [Internet]. 2007 [cited 2025 Sep 30];133:423–32. Available from: https://pubmed.ncbi.nlm.nih.gov/17681163/10.1053/j.gastro.2007.05.029 17681163

[10] Turner D, Griffiths AM, Walters TD, Seah T, Markowitz J, Pfefferkorn M et al. Mathematical weighting of the pediatric Crohn’s disease activity index (PCDAI) and comparison with its other short versions. Inflamm Bowel Dis [Internet]. 2012 [cited 2025 Sep 30];18:55–62. Available from: https://pubmed.ncbi.nlm.nih.gov/21351206/10.1002/ibd.21649 21351206

[11] Koninckx CR, Donat E, Benninga MA, Broekaert IJ, Gottrand F, Kolho KL et al. The Use of Fecal Calprotectin Testing in Paediatric Disorders: A Position Paper of the European Society for Paediatric Gastroenterology and Nutrition Gastroenterology Committee. J Pediatr Gastroenterol Nutr [Internet]. 2021 [cited 2025 Sep 30];72:617–40. Available from: https://pubmed.ncbi.nlm.nih.gov/33716293/10.1097/MPG.0000000000003046 33716293

[12] Andrews S. (2010) FastQC A Quality Control Tool for High Throughput Sequence Data. - References - Scientific Research Publishing [Internet]. [cited 2025 Jun 13]. Available from: https://www.scirp.org/reference/referencespapers?referenceid=2781642

[13] Wingett SW, Andrews S. FastQ Screen: A tool for multi-genome mapping and quality control. F1000Research [Internet]. 2018 [cited 2024 Jun 21];7:1338. 10.12688/f1000research.15931.1 PMC6124377 30254741

[14] Chen S, Zhou Y, Chen Y, Gu J. fastp: an ultra-fast all-in-one FASTQ preprocessor. Bioinformatics [Internet]. 2018 [cited 2024 Jun 13];34:i884–90. Available from: https://pubmed.ncbi.nlm.nih.gov/30423086/10.1093/bioinformatics/bty560 PMC6129281 30423086

[15] Langmead B, Salzberg SL. Fast gapped-read alignment with Bowtie 2. Nat Methods [Internet]. 2012 [cited 2025 Jun 13];9:357–9. Available from: https://www.nature.com/articles/nmeth.1923 10.1038/nmeth.1923 PMC3322381 22388286

[16] Pedregosa FABIANPEDREGOSAF, Michel V, Grisel OLIVIERGRISELO, Blondel M, Prettenhofer P, Weiss R et al. Scikit-learn: Machine Learning in Python Gaël Varoquaux Bertrand Thirion Vincent Dubourg Alexandre Passos PEDREGOSA, VAROQUAUX, GRAMFORT ET AL. Matthieu Perrot. J Mach Learn Res [Internet]. 2011 [cited 2025 Jun 13];12:2825–30. Available from: http://scikit-learn.sourceforge.net

[17] Quast C, Pruesse E, Yilmaz P, Gerken J, Schweer T, Yarza P et al. The SILVA ribosomal RNA gene database project: Improved data processing and web-based tools. Nucleic Acids Res [Internet]. 2013 [cited 2020 Oct 6];41. Available from: https://pubmed.ncbi.nlm.nih.gov/23193283/10.1093/nar/gks1219 PMC3531112 23193283

[18] Davison AC, Hinkley DV. Bootstrap Methods and their Application. Bootstrap Methods their Appl [Internet]. 1997 [cited 2025 Jun 13]; Available from: https://www.cambridge.org/core/books/bootstrap-methods-and-their-application/ED2FD043579F27952363566DC09CBD6A

[19] Canty A, Ripley B. (2022) Boot Bootstrap R (S-Plus) Functions. R Package Version 1.3–28.1. - References - Scientific Research Publishing [Internet]. [cited 2025 Jun 13]. Available from: https://www.scirp.org/reference/referencespapers?referenceid=3500252

[20] Mandrekar JN. Receiver operating characteristic curve in diagnostic test assessment. J Thorac Oncol [Internet]. 2010 [cited 2025 Jun 13];5:1315–6. Available from: https://pubmed.ncbi.nlm.nih.gov/20736804/10.1097/JTO.0b013e3181ec173d 20736804

[21] R Core Team. R: A Language and Environment for Statistical Computing [Internet]. Vienna: R Foundation for Statistical Computing. 2022. Available from: https://www.r-project.org/

[22] Rosen MJ, Dhawan A, Saeed SA. Inflammatory bowel disease in children and adolescents. JAMA Pediatr [Internet]. 2015 [cited 2025 Jun 14];169:1053–60. Available from: https://pubmed.ncbi.nlm.nih.gov/26414706/10.1001/jamapediatrics.2015.1982 PMC4702263 26414706

[23] Cellier C, Sahmoud T, Froguel E, Adenis A, Belaiche J, Bretagne JF et al. Correlations between clinical activity, endoscopic severity, and biological parameters in colonic or ileocolonic Crohn’s disease. A prospective multicentre study of 121 cases. Gut [Internet]. 1994 [cited 2025 Jun 14];35:231–5. Available from: https://pubmed.ncbi.nlm.nih.gov/7508411/10.1136/gut.35.2.231 PMC1374499 7508411

[24] Kittanakom S, Shajib MS, Garvie K, Turner J, Brooks D, Odeh S et al. Comparison of fecal calprotectin methods for predicting relapse of pediatric inflammatory bowel disease. Can J Gastroenterol Hepatol [Internet]. 2017 [cited 2025 Jun 14];2017. Available from: https://pubmed.ncbi.nlm.nih.gov/28491862/10.1155/2017/1450970 PMC5410371 28491862

[25] Shaoul R, Day AS. An Overview of Tools to Score Severity in Pediatric Inflammatory Bowel Disease. Front Pediatr [Internet]. 2021 [cited 2025 Jun 14];9. Available from: https://pubmed.ncbi.nlm.nih.gov/33912519/10.3389/fped.2021.615216 PMC8075054 33912519

[26] Gevers D, Kugathasan S, Denson LA, Vázquez-Baeza Y, Van Treuren W, Ren B et al. The treatment-naive microbiome in new-onset Crohn’s disease. Cell Host Microbe [Internet]. 2014 [cited 2025 Jun 14];15:382–92. Available from: https://pubmed.ncbi.nlm.nih.gov/24629344/10.1016/j.chom.2014.02.005 PMC4059512 24629344

[27] Douglas GM, Hansen R, Jones CMA, Dunn KA, Comeau AM, Bielawski JP et al. Multi-omics differentially classify disease state and treatment outcome in pediatric Crohn’s disease. Microbiome [Internet]. 2018 [cited 2025 Jun 14];6. Available from: https://pubmed.ncbi.nlm.nih.gov/29335008/10.1186/s40168-018-0398-3 PMC5769311 29335008

[28] Alipour M, Zaidi D, Valcheva R, Jovel J, Martínez I, Sergi C et al. Mucosal barrier depletion and loss of bacterial diversity are primary abnormalities in paediatric ulcerative colitis. J Crohn’s Colitis [Internet]. 2016 [cited 2025 Jun 14];10:462–71. Available from: https://pubmed.ncbi.nlm.nih.gov/26660940/10.1093/ecco-jcc/jjv223 PMC4946763 26660940

[29] Shah R, Cope JL, Nagy-Szakal D, Dowd S, Versalovic J, Hollister EB et al. Composition and function of the pediatric colonic mucosal microbiome in untreated patients with ulcerative colitis. Gut Microbes [Internet]. 2016 [cited 2025 Jun 14];7:384–96. Available from: https://pubmed.ncbi.nlm.nih.gov/27217061/10.1080/19490976.2016.1190073 PMC5046168 27217061

[30] Kowalska-Duplaga K, Gosiewski T, Kapusta P, Sroka-Oleksiak A, Wędrychowicz A, Pieczarkowski S et al. Differences in the intestinal microbiome of healthy children and patients with newly diagnosed Crohn’s disease. Sci Rep [Internet]. 2019 [cited 2025 Jun 14];9. Available from: https://pubmed.ncbi.nlm.nih.gov/31827191/10.1038/s41598-019-55290-9 PMC6906406 31827191

[31] Golińska E, Tomusiak A, Gosiewski T, Wiȩcek G, Machul A, Mikołajczyk D et al. Virulence factors of Enterococcus strains isolated from patients with inflammatory bowel disease. World J Gastroenterol [Internet]. 2013 [cited 2025 Jun 14];19:3562–72. Available from: https://pubmed.ncbi.nlm.nih.gov/23801857/10.3748/wjg.v19.i23.3562 PMC3691038 23801857

[32] De Meij TGJ, De Groot EFJ, Peeters CFW, De Boer NKH, Kneepkens CMF, Eck A et al. Variability of core microbiota in newly diagnosed treatment-naïve paediatric inflammatory bowel disease patients. PLoS One [Internet]. 2018 [cited 2025 Jun 14];13. Available from: https://pubmed.ncbi.nlm.nih.gov/30102706/10.1371/journal.pone.0197649 PMC6089417 30102706

[33] Wang X, Xiao Y, Xu X, Guo L, Yu Y, Li N et al. Characteristics of Fecal Microbiota and Machine Learning Strategy for Fecal Invasive Biomarkers in Pediatric Inflammatory Bowel Disease. Front Cell Infect Microbiol [Internet]. 2021 [cited 2025 Jun 14];11. Available from: https://pubmed.ncbi.nlm.nih.gov/34950604/10.3389/fcimb.2021.711884 PMC8688824 34950604

[34] Öhman L, Lasson A, Strömbeck A, Isaksson S, Hesselmar M, Simrén M et al. Fecal microbiota dynamics during disease activity and remission in newly diagnosed and established ulcerative colitis. Sci Rep [Internet]. 2021 [cited 2025 Sep 30];11. Available from: https://pubmed.ncbi.nlm.nih.gov/33883600/10.1038/s41598-021-87973-7 PMC8060394 33883600

[35] Chen L, Lu Z, Kang D, Feng Z, Li G, Sun M et al. Distinct alterations of fecal microbiota refer to the efficacy of adalimumab in Crohn’s disease. Front Pharmacol [Internet]. 2022 [cited 2025 Jun 14];13. Available from: https://pubmed.ncbi.nlm.nih.gov/36034848/10.3389/fphar.2022.913720 PMC9410713 36034848

[36] Vestergaard MV, Allin KH, Eriksen C, Zakerska-Banaszak O, Arasaradnam RP, Alam MT et al. Gut microbiota signatures in inflammatory bowel disease. United Eur Gastroenterol J [Internet]. 2024 [cited 2025 Jun 14];12:22–33. Available from: https://pubmed.ncbi.nlm.nih.gov/38041519/10.1002/ueg2.12485 PMC10859715 38041519

[37] Mancabelli L, Milani C, Lugli GA, Turroni F, Cocconi D, van Sinderen D et al. Identification of universal gut microbial biomarkers of common human intestinal diseases by meta-analysis. FEMS Microbiol Ecol [Internet]. 2017 [cited 2025 Jun 14];93. Available from: https://pubmed.ncbi.nlm.nih.gov/29126267/10.1093/femsec/fix153 29126267

[38] Sabater C, Calvete-Torre I, Ruiz L, Margolles A. Arabinoxylan and Pectin Metabolism in Crohn’s Disease Microbiota: An In Silico Study. Int J Mol Sci [Internet]. 2022 [cited 2025 Jun 14];23. Available from: https://pubmed.ncbi.nlm.nih.gov/35806099/10.3390/ijms23137093 PMC9266297 35806099

[39] Wang F, Kaplan JL, Gold BD, Bhasin MK, Ward NL, Kellermayer R et al. Detecting Microbial Dysbiosis Associated with Pediatric Crohn Disease Despite the High Variability of the Gut Microbiota. Cell Rep [Internet]. 2016 [cited 2025 Jun 14];14:945–55. Available from: https://pubmed.ncbi.nlm.nih.gov/26804920/10.1016/j.celrep.2015.12.088 PMC4740235 26804920

