# Supplementary material for: Tissue and stool microbiome in pediatric inflammatory bowel disease patients: diversity differs in patients with relapsing and non-relapsing Crohn’s disease
Source: Gut Pathog. 2025 Nov 15;17:90. doi: 10.1186/s13099-025-00766-5 (PMC12619421; doi:10.1186/s13099-025-00766-5)
Supplement: Supplementary file 2 — Supplementary Material 2 [file 13099_2025_766_MOESM2_ESM.docx]

**Figure S2:** Multivariate logistic regression for pCD relapse prediction (AUC=1)


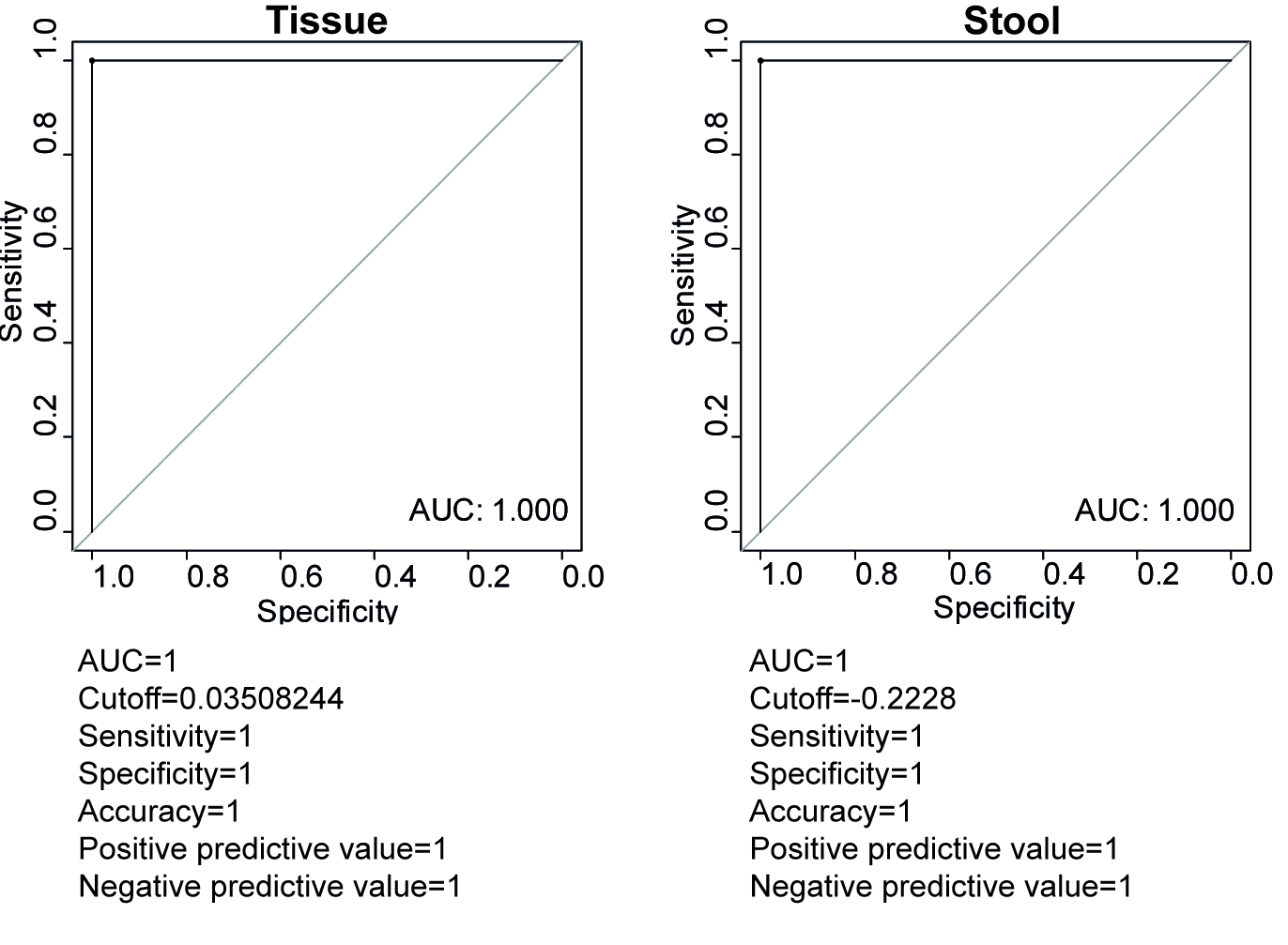


The input data for the multivariate logistic regression included all significantly different taxa with a Log₂ Fold Change > 1, along with richness and wPCDAI values.

The predictive model accurately identified patients at risk of pCD relapse within 12 months after diagnosis, achieving an AUC of 1.

**Tissue model:**

Risk = –527.856309 + 0.124793 × *Morganella* + 102.311292 × *Hydrobacter* + 27.816742 × *Bradyrhizobium* – 26.013712 × *Family_XIII_AD3011_group* – 13.612790 × *Fusicatenibacter* – 6.341383 × *Butyricimonas* + 12.154157 × *Richness*.

**Stool model:**

Risk = 250.643450 + 64.843931 × *Lactobacillus* + 2.358020 × *Lachnospiraceae_UCG-010* + 1.676331 × *Sellimonas* – 10.675158 × *Butyricicoccus*.
