# Supplementary material for: Tissue and stool microbiome in pediatric inflammatory bowel disease patients: diversity differs in patients with relapsing and non-relapsing Crohn’s disease
Source: Gut Pathog. 2025 Nov 15;17:90. doi: 10.1186/s13099-025-00766-5 (PMC12619421; doi:10.1186/s13099-025-00766-5)
Supplement: Supplementary file 3 — Supplementary Material 3 [file 13099_2025_766_MOESM3_ESM.docx]

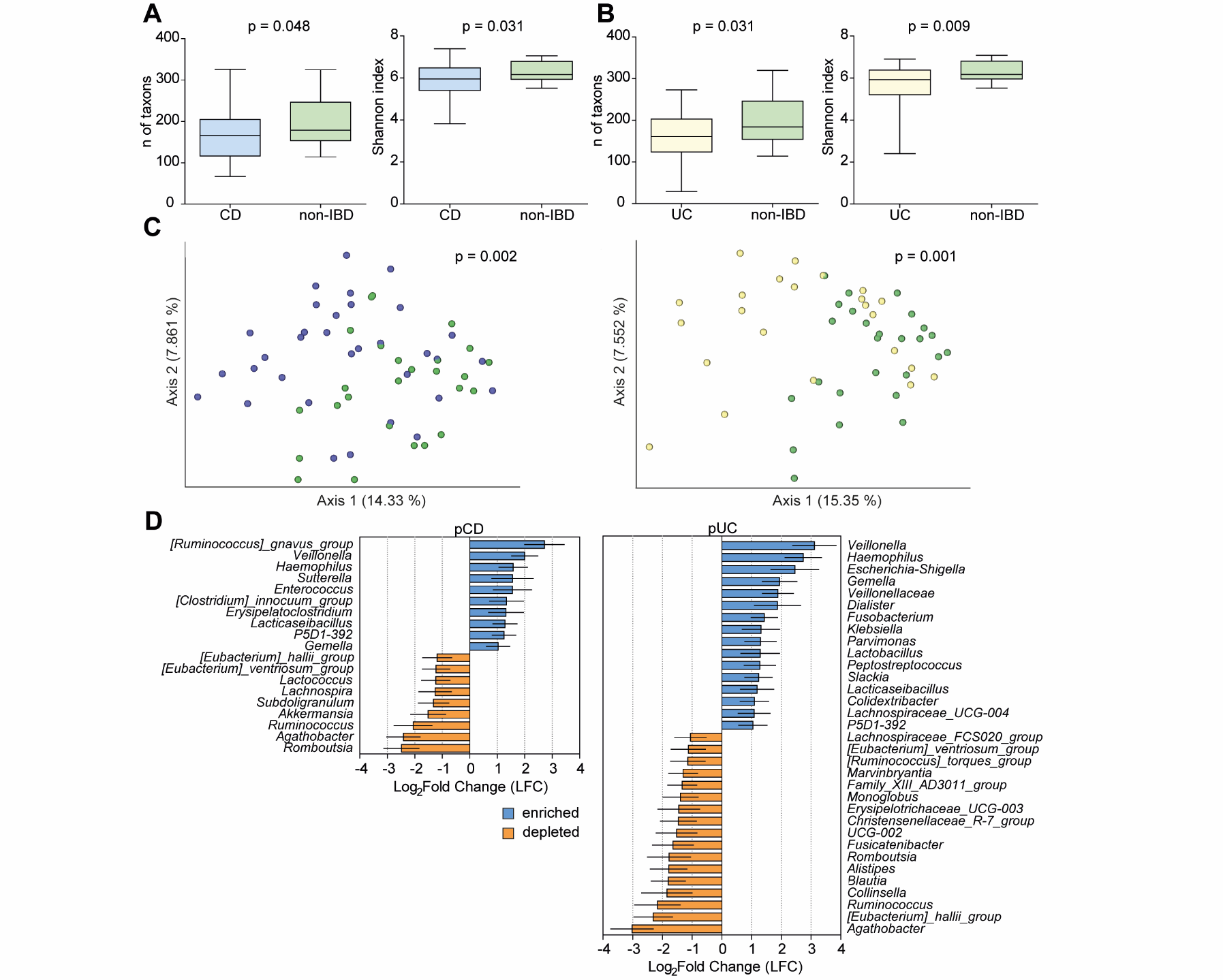


**Supplementary Figure S3:** Microbial diversity and composition in stool samples from patients with pCD (*N* = 33), pUC (*N* = 23), and non-IBD controls (*N* = 26). Taxon richness and the Shannon index were significantly lower in pCD **(A)** and pUC **(B)** compared to non-IBD controls. **(C)** Beta diversity, assessed by unweighted UniFrac, showed significant separation between pIBD patients and non-IBD controls (pCD, blue; pUC, yellow; non-IBD, green). **(D)** Genera significantly enriched (Log_2_ Fold Change (LFC) > 1) and depleted (LFC < -1; p < 0.05) in pCD (left) and pUC (right) groups when compared to controls. Box plots: median, interquartile range (box), min-max range (whiskers). Bars represent the mean of taxa, with error bars showing 95% confidence intervals. Statistical comparisons were performed using the Kruskal-Wallis test for alpha diversity, PERMANOVA for beta diversity, and ANCOM-BC for taxon-level comparisons.
