# Supplementary material for: Tissue and stool microbiome in pediatric inflammatory bowel disease patients: diversity differs in patients with relapsing and non-relapsing Crohn’s disease
Source: Gut Pathog. 2025 Nov 15;17:90. doi: 10.1186/s13099-025-00766-5 (PMC12619421; doi:10.1186/s13099-025-00766-5)
Supplement: Supplementary file 4 — Supplementary Material 4 [file 13099_2025_766_MOESM4_ESM.docx]

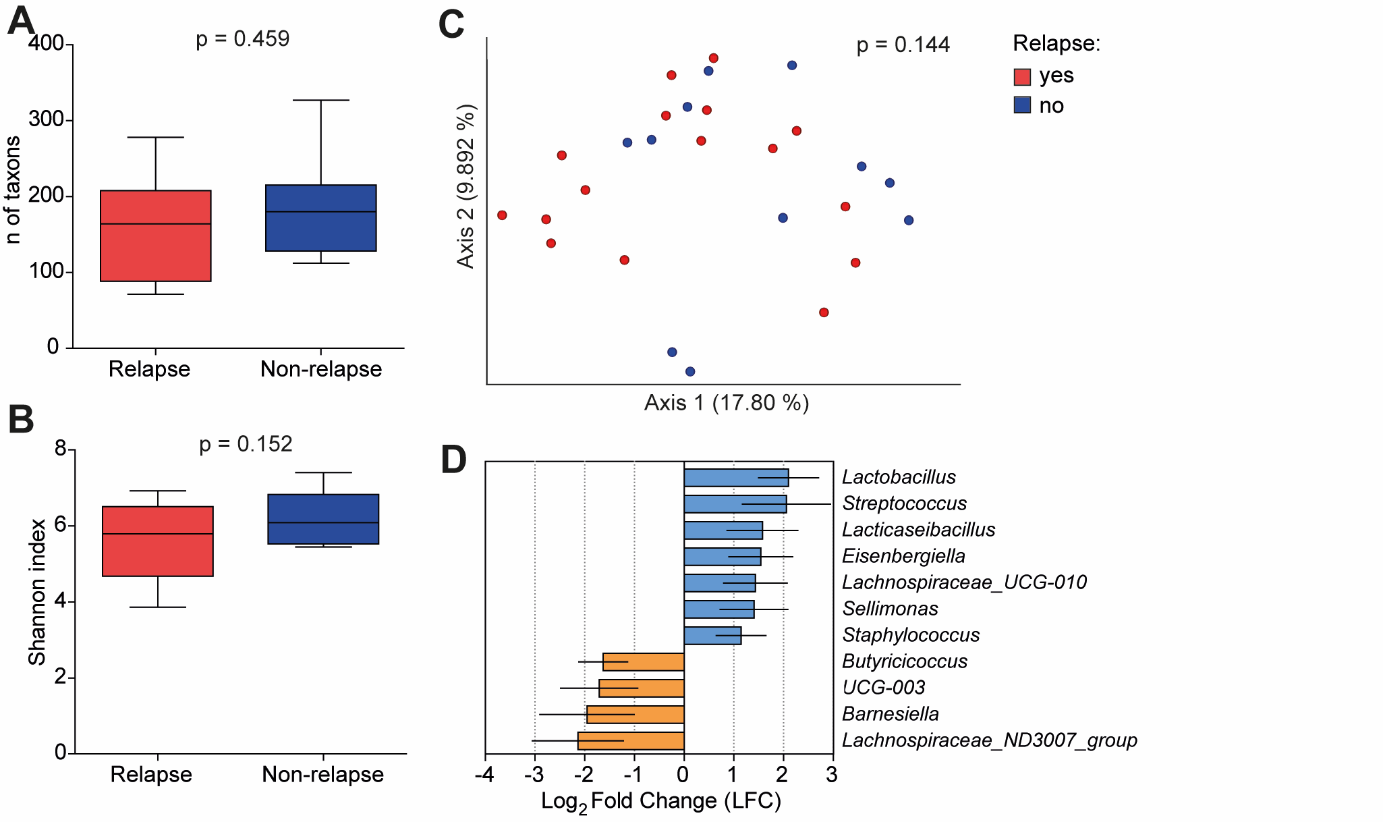


**Supplementary Figure S4:** Microbial diversity and composition in stool samples of pCD patients with relapse (*N* = 16) when compared to non-relapsing patients (*N* = 11). **(A)** Taxon richness and **(B)** Shannon index. **(C)** Beta diversity, assessed by unweighted UniFrac. **(D)** Genera abundance significantly enriched (LFC > 1; p < 0.05) and depleted (LFC < -1; p < 0.05) in pCD patients with relapse. Six pCD patients (out of 33) were excluded from these categories because they did not achieve remission within 12 months. Box plots: median, interquartile range (box), min-max range (whiskers). Bars represent the mean of taxa, with error bars showing 95% confidence intervals. Statistical comparisons were performed using the Kruskal-Wallis test for alpha diversity, PERMANOVA for beta diversity, and ANCOM-BC for taxon-level comparisons.
