# Supplementary material for: Tissue and stool microbiome in pediatric inflammatory bowel disease patients: diversity differs in patients with relapsing and non-relapsing Crohn’s disease
Source: Gut Pathog. 2025 Nov 15;17:90. doi: 10.1186/s13099-025-00766-5 (PMC12619421; doi:10.1186/s13099-025-00766-5)
Supplement: Supplementary file 6 — Supplementary Material 6 [file 13099_2025_766_MOESM6_ESM.docx]

|  | **Parameter** | **AUC** |
| --- | --- | --- |
|  | wPCDAI | 0.744 |
| **tissue** | Richness | 0.761 |
|  | Shannon index | 0.767 |
|  | *Morganella* | 0.594 |
|  | *Hydrobacter* | 0.688 |
|  | *Bradyrhizobium* | 0.614 |
|  | *P5D1-392* | 0.727 |
|  | *Family_XIII_AD3011_group* | 0.722 |
|  | *Fusicatenibacter* | 0.733 |
|  | *UBA1819* | 0.719 |
|  | *Anaerostipes* | 0.739 |
|  | *UCG-003* | 0.713 |
|  | *Christensenellaceae_R-7_group* | 0.733 |
|  | *Subdoligranulum* | 0.739 |
|  | *Parabacteroides* | 0.747 |
|  | *Butyricimonas* | 0.790 |
|  | *Collinsella* | 0.773 |
|  | *Barnesiella* | 0.818 |
| **stool** | Richness | 0.585 |
|  | Shannon index | 0.665 |
|  | *Lactobacillus* | 0.750 |
|  | *Streptococcus* | 0.733 |
|  | *Lacticaseibacillus* | 0.653 |
|  | *Eisenbergiella* | 0.676 |
|  | *Lachnospiraceae_UCG-010* | 0.594 |
|  | *Sellimonas* | 0.594 |
|  | *Staphylococcus* | 0.625 |
|  | *Butyricicoccus* | 0.727 |
|  | *UCG-003* | 0.693 |
|  | *Barnesiella* | 0.696 |
|  | *Lachnospiraceae_ND3007_group* | 0.716 |

**Supplementary Table S3:** Receiver Operating Characteristic (ROC) curve analysis: Area under the curve for pediatric Crohn's disease patients experiencing relapse
